# The Danish Symptom Cohort: Questionnaire and Feasibility in the Nationwide Study on Symptom Experience and Healthcare-Seeking among 100 000 Individuals

**DOI:** 10.1155/2014/187280

**Published:** 2014-07-23

**Authors:** Sanne Rasmussen, Jens Søndergaard, Pia Veldt Larsen, Kirubakaran Balasubramaniam, Sandra Elnegaard, Rikke Pilsgaard Svendsen, Rikke Sand Andersen, Anette Fischer Pedersen, Peter Vedsted, Dorte Ejg Jarbøl

**Affiliations:** ^1^Research Unit of General Practice, Institute of Public Health, University of Southern Denmark, J. B. Winsløws Vej 9A, 5000 Odense C, Denmark; ^2^The Research Unit for General Practice, Danish Research Centre for Cancer Diagnosis in Primary Care (CaP), Department of Public Health, Aarhus University, Bartholins Allé 2, 8000 Aarhus C, Denmark

## Abstract

*Introduction*. In order to develop strategies to prevent delay in diagnosis, it is important to gain knowledge of symptoms and healthcare-seeking processes in the population. This paper describes a combined survey and register-based study with (1) focus on development of a questionnaire concerning experience of symptoms and subsequent consequences and (2) feasibility of the study. *Methods*. The study is a nationwide cohort study of 100 000 individuals randomly selected from the Danish general population. A comprehensive questionnaire concerning experience of symptoms and subsequent consequences was developed. The methodological framework for the development included defining the domains to be measured, identification of previous items, scales and questionnaires in the literature, and pilot and field testing. *Results*. A total of five domains and 16 subdomains were defined covering the area of symptom experience, symptom characteristics, reaction in response to symptom experience, external factors, and personality characteristics with potential influence on the symptom experience. In total, 49 706 questionnaires were completed, yielding a response rate of 52.2%. *Conclusion*. We developed a comprehensive questionnaire used in a large combined survey and register-based study concerning experience of symptoms and subsequent consequences of symptom experiences. We succeeded in conducting a large survey providing the groundwork for The Danish Symptom Cohort.

## 1. Introduction

Symptom experiences among people in the general population are common. Studies have shown that over three quarters of adults have experienced a symptom within the past two weeks, and up to 15% have experienced an alarm symptom of one of the four most frequently occurring cancers within the preceding year [1, 2].

Symptom experiences are embedded in a complex interplay of biological, psychological, and cultural factors and may reflect a variety of interpretations of sensations, which are not necessarily expressions of underlying disease. Many people manage symptom experiences privately without consulting the healthcare system. Thus only some symptom experiences are presented to the healthcare system, a phenomenon referred to as the “symptom iceberg” [3, 4]. From a medical perspective some of these symptoms might be alarming and indicating serious disease that ought to be examined by a physician. The issue of proper healthcare-seeking is often raised in connection with serious diseases, in particular in terms of patients' awareness and knowledge of symptoms and the “patient interval,” that is, the time elapsed from a patient experiencing a symptom until seeking healthcare [5].

Some of the factors which can affect the decision to seek healthcare are symptom characteristics, impact on daily activities, personal experience with or witnessing of symptoms and disease, health perceptions, and network and social context [1, 6–8]. In spite of the existing literature on possible determinants of healthcare-seeking, understanding of symptom experience, interpretation, and management in everyday life is still deficient. Most of the existing research is based on retrospective studies on selected groups of patients making it difficult to generalize to the general population [5, 7].

Providing timely and effective healthcare in relation to serious diseases is an important task for the healthcare system. In order to develop strategies to prevent delayed diagnosis, it is important to gain knowledge of the symptoms occurring in the general population and knowledge about factors important for the healthcare-seeking process. The purpose of this paper is to describe the development of a comprehensive questionnaire concerning experience of symptoms, interpretation, and subsequent consequences of symptom experiences. The specific objectives are (1) conceptualization of “symptom experience” and “healthcare-seeking,” defining the domains to be measured, identification and inclusion of previously used and validated items, scales, and questionnaires, and development of single symptom items pilot and field testing of the comprehensive questionnaire and (2) the feasibility of the study.

## 2. Methods

The study was designed as a nationwide cohort study of 100 000 people randomly selected from the general population and with baseline data collected in a web-based survey. All Danish citizens are registered in the Danish Civil Registration System (CRS) with a unique personal identification number enabling accurate linkage between national registers. In Denmark researchers are prohibited by law to contact persons who have declined to participate in research-related inquiries. These persons were excluded prior to sampling, based on information in the CRS. From the CRS, 100 000 adults aged 20 years or above were randomly selected and invited to participate in the survey. The individuals received a postal letter explaining the purpose of the study. In the letter a unique 12-digit login for a secure webpage was included.

### 2.1. The Study Population, Recruitment, and Logistics

Due to the large sample size the study population was randomly divided into four equally sized groups. The groups were invited with two-week time intervals, except the first two groups who were invited with a four-week interval because of the holiday season. A reminder letter followed the initial invitational letter to nonrespondents after two weeks. After additional two weeks the nonrespondents were contacted by telephone and encouraged to participate. A private telemarketing company conducted the latter reminder procedure. In this procedure the participants were offered the opportunity to receive an e-mail with a direct link to the questionnaire.

In order to prevent the exclusion of people with no access to a computer, tablet, or smartphone we offered the participants the opportunity of the survey being conducted as a telephone interview. A group of trained interviewers were assigned to conduct the telephone interviews. The interviewers were instructed to read the questions and prespecified options out loud, not contributing with their own interpretation of the questions. We drew lots for 10 iPads among all the respondents as gratuity for their efforts.

### 2.2. Electronic Platform

The questionnaire was designed in the web-based online platform SurveyXact [9]. To access the questionnaire, the respondent had to open a webpage and enter the provided 12-digit login. The electronic format of the questionnaire made it possible to construct a leap structure, so the respondents were directed through the questionnaire according to the answers already given and hereby skipping irrelevant questions.

### 2.3. Developing the Questionnaire

The conceptual objective of the study was to measure the prevalence of symptom experience in the general population and the individual's interpretation of symptoms and subsequent healthcare-seeking behaviour. Data concerning self-reported experience of symptoms and subsequent consequences together with personal and social characteristics were collected using a comprehensive questionnaire. The questionnaire is based on standard rating scales, previously validated questionnaires, and ad hoc items.

The methodological framework for developing the questionnaire included the following steps [10]:

1. based on the objective, defining the constructs and conceptual framework, and exploring the literature on these concepts with the purpose of defining the domains to be measured;
2. identification of previous items, scales, and questionnaires in the literature followed by item generation and item reduction;
3. pilot testing with regard to content validity, relevance, acceptability, and feasibility;
4. field testing within a pilot study on 500 randomly selected people from the general population testing data quality and floor and ceiling effects and assessing the distribution of the answers in the various categories. Besides field testing the pilot study was used to test study procedures, resources, time scale, and estimation of the recruitment rate.

#### 2.3.1. Defining the Constructs and Conceptual Framework and Exploring the Literature regarding Measuring Instruments Fulfilling the Objective

The first step in the process was to define the concepts of “symptom experience” and “healthcare-seeking behaviour.”

The term symptom as presented in the discipline of medicine is influenced by a desire to predict underlying diseases and risks of negative effects on health. In medicine, it is common to distinguish between subjective health complaints (symptoms) and signs with the latter being objectively verifiable (e.g., blood in the urine or jaundice), whereas symptoms often refer to subjective complaints. The WONCA Dictionary of General/Family Practice defines a symptom as “any subjective evidence of a health problem as perceived by the patient” [11]. The fact that symptoms are subjective complaints should be emphasised when exploring symptom experiences. In this study, we define subjectively reported symptom experiences as multidimensional constructions embedded in a complex interplay of biological, psychological, and cultural factors. This definition implies that symptom experiences are not viewed as objective, clinical phenomena but are seen as the result of the patient's own interpretation process, in which bodily sensations or changes are transformed into signs of ill health [6, 12, 13].

Current literature on healthcare-seeking often presents a dichotomous approach: either you seek medical advice or you do not seek medical advice. In this study we wanted to approach healthcare-seeking behaviour in a multifactorial context [14]. A group of experts from different disciplines in symptom research was established. The group comprised a psychologist, an anthropologist, and six medical doctors. Based on the literature from the three different disciplines and discussions in the group, the constructs “symptom experience” and “healthcare-seeking behaviour” were defined and a conceptual framework for both constructs was formed, resulting in a combined formative and reflective model.

*The Developing Process of the Questionnaire.* The developing process involved several meetings in the group comprising the three different disciplines: psychology, anthropology, and medical science. The development of the domains as well as the item generation in each domain was based on the literature of symptoms and healthcare-seeking and related factors. Overall the discussion in the group concerned (1) reflections on which symptoms to include where the emphasis was on frequency and severity, (2) recall periods, and (3) operationalising the conceptual framework. 

*Domains in the Questionnaire*. Based on our conceptual framework, a core content of domains was developed to be included in the questionnaire. A total of five domains were identified (Table 1).

Three domains consider the symptom experience and actions taken in response to the symptom experience.

- The first domain addresses the occurrence of different “symptom experiences” during the preceding four weeks.
- The second domain concerns symptom experience characteristics in terms of time point for first symptom experience, concerns, and impact on activities of daily living.
- The third domain explores the reactions and actions taken in response to symptom experiences, for example, discussion with friends and family, healthcare-seeking behaviour, and potential barriers to seeking medical advice.

The remaining two domains were included in order to explore how various factors may influence symptom experience and healthcare-seeking.

(iv) The fourth domain concerns the potential influence of a number of selected factors on the symptom experience, for example, lifestyle, social network, the GP's and the surroundings' reactions to—and concern for—the person's health, and experience of illness in the immediate family and circle of friends.
(v) The fifth domain considers the respondents' personality characteristics such as self-rated health, coping strategies, and their attitude towards risk taking.

#### 2.3.2. Item Generation and Item Reduction

*Domain 1: Symptom Experience.* We created a large item bank with various predefined symptoms. In relation to the conceptual framework we aimed to include a variety of bodily symptom experiences. From a medical perspective some of these are categorized as alarm symptoms, but also a variety of symptoms that are frequently occurring and categorized as benign were included. The following subdomains were identified: (1) specific and nonspecific cancer alarm symptoms, (2) general, frequent symptoms, (3) abdominal symptom complexes, and (4) bodily distress syndrome.

Re 1. For representativeness of symptoms that from a medical perspective are defined as indicating a serious disease, we selected a number of specific and nonspecific alarm symptoms covering the following areas: lung, gastrointestinal, gynaecological, and urogenital cancer. The symptoms were selected based on a review of literature, national and international cancer referral guidelines, and descriptions of cancer pathways [15–17].

Re 2. We included a number of general symptoms based on the knowledge that cancer patients in addition to presenting a specific alarm symptom often also present vague and uncharacteristic symptoms [15, 18]. The selection of these symptoms was based on retrospective studies of cancer patients' registration of symptoms prior to their first contact [5, 7].

Re 3. As an example of an organ-specific area, we chose abdominal symptoms, because abdominal symptoms are frequently occurring in the population, are often occurring in frequent healthcare-seekers, and are associated with a range of definable symptom-based conditions, for example, dyspepsia, gastroesophageal reflux disease, and irritable bowel syndrome. Further, there are symptoms which both occur in cancer referral pathways and in the functional abdominal symptom complexes. The selection of symptoms was based on the latest symptom-based consensus classification regarding dyspepsia and irritable bowel syndrome (the ROME III criteria) [19–21], while the symptoms forming the basis for diagnosis of reflux disease were defined by the Montreal criteria [22].

Re 4. Symptoms not attributable to any medical diagnosis challenge the concept of diagnosis. Patients with symptom experiences from numerous organ systems and with a high symptom burden are referred to in a number of different ways in the literature, for example, unexplained medical disorders, psychosomatic disease, or somatisation. These symptoms are now assembled in one classification called bodily distress syndrome (BDS). Items already identified to classify BDS were included [23].

Based on the subdomains, we created items asking whether people had experienced one or more of the predefined symptoms within the preceding four weeks.

*Domain 2: Symptom Experience Characteristics*. In the second domain we wanted to explore the time point for each symptom experience, concerns in relation to the symptom experience, and impact on activities of daily living.

People are more likely to seek healthcare if symptoms are perceived as severe or incapacitating [3]. The persistence of a symptom may also influence the way people interpret it differently [24]. The literature emphasises symptom severity as a phenomenon that should involve integration of patient-reported severity ratings in combination with clinical measures, such as daily functional status or concurrent psychosocial features [25, 26]. Therefore, items regarding when the symptoms were experienced for the first time and how the symptoms had influenced the respondents' daily activities were included. Additionally, an item asking whether the respondents had had concerns regarding their symptom experience was included.

*Domain 3: Reactions in Response to Symptom Experience*. Studies have shown that use of and access to social network, for example, telling and interacting with others when experiencing symptoms or asking for advice, influences healthcare-seeking [27]. Viewed from a psychological perspective the use of social network in relation to illness and stressful situations is often characterized by the following two types of social support: instrumental support in terms of practical help, advice, and emotional support in the form of confidentiality, empathy, and love [28]. The use of social network in relation to help and healthcare-seeking is from a social science viewpoint based on the theory that people generally neither make a single choice nor plan a set of choices. When experiencing potential signs of illness, they will ask for advice and seek help from a wide variety of lay, professional, and semiprofessional others until the situation is resolved or options are exhausted [26]. In combining social science and psychological approaches we therefore developed items on whether the respondents had consulted their family, friends, coworkers, and so forth and/or their GP, other doctors, community nurses, physiotherapists, and so forth when experiencing a symptom.

Studies have shown that people often experience one or more barriers to seek healthcare. The respondents were asked to decide on four of the most common considerations in relation to healthcare-seeking [29]. Moreover, we included a free text box encouraging the respondents to add additional considerations regarding contact to their GP in relation to every symptom experience.

*Domain 4: Selected Factors with Potential Influence on the Symptom Experience and Healthcare-Seeking*. The fourth domain was developed based on the hypothesis that the symptom experience and healthcare-seeking behaviour may be influenced by a number of external factors. We therefore included items from existing questionnaires on lifestyle factors, for example, smoking and alcohol consumption, and items about general contact to the social network as well as the GP's and social network's expressed concern for the respondent's health. Moreover, we included items exploring experience of illness in the immediate family and circle of friends [30].

*Domain 5: Personality Characteristics in relation to Symptom Experience and Healthcare-Seeking*. One of the aims of the study is to examine possible associations between personality facets, symptom experiences, and healthcare-seeking behaviour, respectively. Risk-taking attitudes as well as dispositional coping strategies have shown to be associated with various types of health-related behaviours in different patient populations [31–35]. In the fifth domain we therefore included a number of items on self-rated health and attitude towards risk-taking. Additional to these items, we included a scale assessing the prevailing tendency to use approach or avoidant coping strategies. The coping scale was available only in English and was therefore translated into Danish according to standardised methods [36].

On the last page of the questionnaire we added a commentary box encouraging respondents to add additional comments and to report any comprehension problems in the questionnaire.

#### 2.3.3. Pilot Testing

The aim of the pilot testing was to test the content validity, that is, relevance, acceptability, and feasibility. Before conducting the first test, the questionnaire was discussed by a multidisciplinary expert panel comprising a psychologist, an anthropologist, a biostatistician, two GPs, and five medical doctors. Further, the questionnaire was sent out to a group of 25 persons from an academic setting (all researchers in healthcare, natural sciences, and humanities) and was further discussed in this academic setting. This resulted in both alterations in the order of the items and clarification of introductory captions to each of the domains.

The pilot testing was conducted in two stages, the first with seven women and four men aged 22–61 years who were observed as they completed the questionnaire. The people participating in the pilot testing were recruited at a train station outside a public library. Trained interviewers asked the participants to read each item and answer it while thinking out loud, highlighting problems, and expressing their attitude to the question.

The second round of pilot testing included 11 new participants, four men and seven women, with an age range of 20–78 years.

#### 2.3.4. Field Testing

A pilot study was conducted among 500 Danish adults aged at least 20 years and randomly selected using the CRS. We used the electronic platform SurveyXact and offered the possibility of completing the questionnaire by a telephone interview. Field testing of the questionnaire was incorporated in the pilot study. We assessed the mean and standard deviation of each item, median, and extent of floor and ceiling effects.

## 3. Ethics 

The Regional Scientific Ethics Committee for Southern Denmark evaluated the project and concluded that the project was not notifiable and could be implemented without the permission from The Regional Scientific Ethical Committee for Southern Denmark according to Danish law. The participants in the study were clearly informed that there would be no clinical follow-up and that they should contact their own GP in case of concern or worry. The project has been approved by the Danish Data Protection Agency (journal number 2011-41-6651).

## 4. Results

### 4.1. Development of the Questionnaire

A conceptual framework with regard to symptom experience and healthcare-seeking was developed. The final questionnaire consisted of five domains with 16 subdomains. Table 1 shows the domains and subdomains. A total of 44 single symptoms were included (Table 2). Content validity, that is, relevance, acceptability, and feasibility of the comprehensive questionnaire, was assessed in the pilot testing. The first round of pilot testing identified items where the participants' understanding differed from the intended meaning as well as items causing confusion or uncertainty. This stage of pilot testing resulted in alterations in the caption of the first domain. The second round resulted in alterations in layout and wordings and exclusion of items with poor feasibility, for example, items about “worrying about illnesses” which the participants found difficult and aggravating.

Field testing of the questionnaire was performed in a pilot study on 500 people resulting in alterations in formulations of questions and introductory captions.

### 4.2. Feasibility of the Study

A pilot study was conducted as a part of the feasibility assessment. In the pilot study a total of 182 of the 500 subjects completed the questionnaire, yielding a response rate of 36.4%. The final questionnaire was adjusted in light of the pilot and field testing. Changes primarily consisted of alterations in formulations of questions and introductory captions.

The response rate of 36.4% in the pilot study resulted in the hiring of a telemarketing company to perform reminder phone calls. The pilot study also provided an estimate on how many telephone interviews as well as incoming calls with questions from the respondents would be expected.

**Figure 1:**
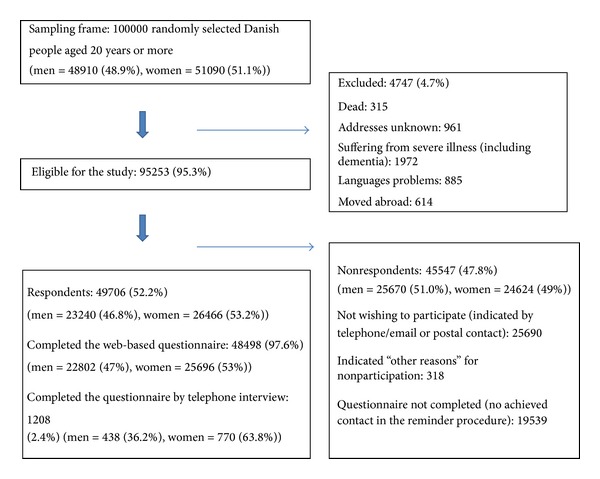
Study cohort.

Of the 100 000 randomly selected subjects, 4 474 (4.7%) were not eligible because they had either died, could not be reached due to unknown address, were suffering from severe illnesses (including dementia), had language problems, or had moved abroad. Information on severe illness and subjects who had moved abroad was obtained through contact with family or relatives in the reminder procedure. Of the 95 253 (95.3%) eligible subjects, 49 706 subjects completed the questionnaire, yielding an overall response rate of 52.2% (Figure 1). The median age of the respondents was 52-year IQR (40–64) compared to 50-year IQR (36–67) for nonrespondents.

The pilot study provided information with regard to the logistics resulting in the division of the study into four waves.

Table 3 shows the results of the four waves in terms of the number of invited participants in each wave and the cumulated response rate after the invitational letter (30.4%), after the reminder letter (43.7%), and after telephone reminder (52.2%), respectively. In addition, Table 3 shows the response rate for telephone interviews and the age and gender distribution.

## 5. Discussion

### 5.1. Measuring Symptom Experiences

Well-known medical symptoms and bodily sensations including cancer alarm symptoms formed the base of the questionnaire. The survey was based on a limited number of predefined symptoms, leaving some respondents in a situation where they could not express all their symptoms. Instead, we opted to gather a more comprehensive analysis of the impact of the symptom on functioning and how the respondent's social network is utilized with respect to the symptom. To allow respondents to elaborate on their symptom experience in their own words and to learn more about the respondents' understanding of symptoms, we added a commentary box encouraging respondents to add additional comments, creating a possibility of evaluating the comprehension of the questionnaire.

In designing the questionnaire we included anthropological, psychological, and medical paradigms. To our knowledge no previous surveys on symptoms have used a similar multiperspective approach. Using this approach, we believe that symptom experiences in the general population can be analysed in a more nuanced way and with greater precision. In an attempt to minimise recall bias, we chose to ask about symptoms experienced within the last four weeks.

### 5.2. Response Rate and Participant Characteristics

The large sample of 100 000 subjects with 49 706 completed baseline questionnaires makes it possible to estimate prevalence of, for example, cancer alarm symptoms and of some rarer conditions with good statistical precision.

The subjects invited to participate in the study were randomly selected using the Danish Civil Registration System, which reduces the risk of selection bias. The response rate in the study (52.2%) was slightly lower than in previous Danish population-based surveys [37]. This could be due to the fact that the questionnaire was web-based and not available in hard copy. However, the invitational letter did encourage people with no access to computer, tablet, or smartphone to contact us by telephone for the possibility of completing the survey by telephone interview.

### 5.3. Telephone Interviews

A minority of the respondents (2.4%) responded to the questionnaire via telephone interview. It is possible that data from the interviews are different from the data derived from the online version. The difference was sought minimised by using trained interviewers who were instructed only to read the questions out loud and ask the respondent to choose between the available answers. The interviewers were explicitly instructed not to engage in the interpretation of the questions with the respondent. However, the fact that the telephone respondents were not able to see the questions and answers themselves and reflect on the answers in private might have affected their answers. The respondents who participated by telephone interview were mainly women in the older age groups. Obtained data may differ between self-administered questionnaires and telephone interviews. It is, however, not possible to determine whether differences would be caused by selection due to variations in age, sex, health status, or socioeconomic position or whether the differences would be due to the answering method.

### 5.4. Feasibility of the Study

We succeeded in conducting a large survey providing the groundwork for The Danish Symptom Cohort. Conducting the study in four waves solved the logistic challenges in terms of making it possible to handle both incoming calls with questions and conducting telephone interviews. The use of a telephone reminder procedure was implemented due to the poor response rate in the pilot study. The increase in the response rate of 8.5 percentage points from the reminder letter to the telephone reminder was a result in line with what we had expected. A similar procedure may well be applied in other large population-based studies.

### 5.5. Linkage to Register Data

To explore possible selection bias linkage to register data allows us to compare respondents and nonrespondents with regard to sex, age, socioeconomic status, and healthcare utilisations. Moreover, we are able to compare early and late respondents based on available information on the distribution date of the questionnaire and on the date the questionnaire was filled in.

### 5.6. Implications for Research and Practice

Early diagnosis and prompt treatment are generally presumed to be a key to a better prognosis of most illnesses. To improve early diagnosis it is important to gain knowledge about which symptoms, groups of symptoms, and related factors lead to healthcare-seeking. Information from this study will provide important knowledge on symptom experiences as well as on relationships between specific symptom experiences and, respectively, personal characteristics, subsequent healthcare-seeking, and the patient's interaction with the healthcare system. This large cohort of individuals will be followed for a number of years with complete follow-up on development of diseases by linkage with register data on socioeconomic status, health care utilisation, and hospital based diagnosis. By gaining this knowledge it will be possible for healthcare providers to form better and more precise referral pathways and hereby minimising delay in diagnosis.

## 6. Conclusion

We developed a comprehensive questionnaire consisting of five domains with 16 subdomains covering the area of symptom experience, symptom characteristics, reaction in response to symptom experience, external factors, and personality characteristics with potential influence on the symptom experience. We succeeded in conducting a large survey providing the groundwork for The Danish Symptom Cohort. With 49 706 having completed the questionnaire, an overall response rate of 52.2% was achieved.

## Figures and Tables

**Table 1:** Domains in the questionnaire.

Domains	Subdomains	Items
(1) Symptom experience	Specific and nonspecific cancer alarm symptoms	Alarm symptoms covering the following areas
		Lung, gastrointestinal, gynaecological, and urogenital cancer
		General, frequent symptoms
	Fever	
	Feeling unwell	
	Feeling ill	
	Loss of appetite	
	Abdominal symptom-based conditions	Symptoms based on the consensus classification regarding dyspepsia and irritable bowel syndrome (the Rome III criteria)
		Symptoms forming the basis for diagnosis of gastroesophageal reflux disease (Montreal criteria)
	Bodily distress syndrome	Items identified to classify BDS, covering the following areas
		Cardiopulmonary
		Gastrointestinal
		Musculoskeletal
		General symptoms

(2) Symptom experience characteristics	Debut	First occurrence of the symptom experience
	Impact on daily life	Impact of the symptom experience on daily life activities
	Concerns	The respondents' concerns regarding the symptom experience

(3) Reactions in response to symptom experience	Use of social network	Discussion of symptom experience with friends, acquaintances, and family
		Contact with the GP, other therapists, hospital doctors, community nurses, physiotherapists, etc.
	Considerations about contact with the GP	Decisions of four barriers towards healthcare-seeking using the ABC (awareness and beliefs about cancer)
		“It would be embarrassing for me”
		“I would be worried about wasting the doctor's time”
		“I would be worried about what the doctor might find”
		“I was too busy to find time to go to the doctor”

(4) Selected factors with potential influence on the symptom experience and healthcare-seeking	Life style	Smoking
		Alcohol consumption
		Body mass index
	Access to social network	Frequency of contact in terms of telephone conversations and/or communion with others
	Concerns for the respondent	The GP's and surroundings' reaction and concern for the respondent
	Experience with illness	Experience with serious illness in the immediate family and close friends

(5) Personality characteristics in relation to symptom experience and healthcare-seeking	Attitude towards risk-taking	Attitude towards risk in relation to health and finances
	Self-rated health	Respondents assessment of own health
	Coping strategies	Respondents assessments of coping with problems using The Brief Approach/Avoidance Coping Questionnaire

**Table 2:** The 44 included single symptom experiences.

Tiredness	Erectile dysfunction
Night-time urination	Pelvic pain
Lack of energy	Shortness of breath
Headache	Hoarseness
Back pain	Urge incontinence
Abdominal bloating	Loss of appetite
Memory problems	Blood in stool/rectal bleeding
Abdominal pain	Pelvic pain during intercourse
Coughing	Fever
Concentration problems	Difficulty swallowing
Change in stool texture	Weight loss
Dizziness	Incontinence without stress/urge
Feeling unwell	Pain/burning when urinating
Constipation	Lump/swollen lymph node
Increase in waist circumference	Black stool
Change in stool frequency	Repeated vomiting
Diarrhea	Vaginal bleeding after intercourse
Nausea	Postmenopausal bleeding
Swollen legs	Blood in urine
Difficulty in emptying the bladder	Blood in semen
Frequent urination	Coughing up blood
Stress incontinence	Blood in vomit

**Table 3:** Results from the four waves.

	Wave 1	Wave 2	Wave 3	Wave 4	Total
*Invited for participation *	25 000	25 000	25 000	25 000	100 000
Men (%)	12 203 (48.8)	12 180 (48.7)	12 348 (49.4)	12 179 (48.7)	48 910 (48.9)
Women (%)	12 797 (51.2)	12 820 (51.3)	12 652 (50.6)	12 821 (51.3)	51 090 (51.1)

*Dead (%) *	25 (0.1)	78 (0.3)	85 (0.3)	127 (0.5)	315 (0.3)
*Addresses unknown (%) *	247 (1.0)	252 (1.0)	236 (0.9)	226 (0.9)	961 (1.0)
*Suffering from severe illness (including dementia) (%) *	556 (2.2)	480 (1.9)	468 (1.9)	468 (1.9)	1 972 (2.0)
*Language problems (%) *	233 (0.9)	228 (0.9)	195 (0.8)	229 (0.9)	885 (0.8)
*Moved abroad (%) *	53 (0.2)	172 (0.7)	188 (0.8)	201 (0.8)	614 (0.6)
*Excluded (% of invited) *	1 114 (4.5)	1 210 (4.8)	1 172 (4.7)	1 251 (5.0)	4 747 (4.7)

*Eligible for the study (% of invited) *	23 886 (95.5)	23 790 (95.2)	23 828 (95.3)	23 749 (95.0)	95 253 (95.3)
*Respondents (%) *	12 361 (51.7)	12 763 (53.6)	12 298 (51.6)	12 284 (51.7)	49 706 (52.2)
Men (%)	5 718 (46.3)	5 911 (46.3)	5 815 (47.3)	5 796 (47.2)	23 240 (46.8)
Women (%)	6 643 (53.7)	6 852 (53.7)	6 483 (52.7)	6 488 (52.8)	26 466 (53.3)
Median age (IQR)	52 (40–64)	52 (40–64)	52 (40–64)	52 (39–64)	52 (40–64)

*Completed by telephone interview (% of respondents) *	342 (2.8)	308 (2.4)	286 (2.3)	272 (2.2)	1 208 (2.4)
Men (%)	129 (37.7)	110 (35.7)	93 (32.5)	106 (39.0)	438 (36.3)
Women (%)	213 (62.3)	198 (64.3)	193 (67.5)	166 (61.0)	770 (63.7)
Median age (IQR)	74 (67–79)	74 (68–96)	73 (68–80)	74 (68–94)	74 (68–96)

*Completed after initial letter (% of respondents, cumulative) *	6 437 (26.9)	7 583 (31.9)	7 559 (31.7)	7 353 (31.0)	28 932 (30.4)
*Completed after reminder letter (% of respondents, cumulative) *	3 925 (43.4)	3 130 (45.0)	2 713 (43.1)	2 908 (43.2)	12 676 (43.7)
*Completed after telephone reminder (% of respondents, cumulative) *	1 999 (51.7)	2 050 (53.6)	2 026 (51.6)	2 023 (51.7)	8 098 (52.2)

*Nonrespondents (% of eligible) *	11 525 (48.3)	11 027 (46.4)	11 530 (48.4)	11 465 (48.3)	45 547 (47.8)
Not wishing to participate (indicated by telephone/email or postal contact) (%)	6 880 (28.8)	6 287 (26.4)	6 407 (26.9)	6 116 (25.8)	25 690 (27.0)
Indicated “other reasons” for nonparticipation (%)	11 (0.04)	85 (0.4)	99 (0.4)	123 (0.5)	318 (0.3)
Nonrespondents (no achieved contact in the reminder procedure) (%)	4 634 (19.4)	4 655 (19.6)	5 024 (21.1)	5 226 (22.0)	19 539 (20.5)
